# Supplementary material for: Amnat Charoen Healers in Thailand and Their Medicinal Plants
Source: Plants (Basel). 2025 Feb 17;14(4):602. doi: 10.3390/plants14040602 (PMC11859466; doi:10.3390/plants14040602)
Supplement: Supplementary file 1 [file plants-14-00602-s001.zip › plants-3399984-supplementary.pdf]

Appendix S1. Medicinal plants used by fifteen Phu Tai healers in Amnat Charoen province, Thailand.
(Life form Habit and Status; CR = Critically Endangered, E=Endemic, EN=Endangered, H=Herb, HC= Herb Climber, R= Rare, NT = Near Threatened, S= Shrub, ST=Shrub Tree, US= Under Shrub, T=Tree, C=Climber; P= Palm; Native or exotic: Ex=Exotic; *Methods and Applications;* Ext= External, In= Internal). The Original data or native rank of exotic medicinal plants in this study according on POWO.

Family	Scientific names (Vourcher no.)	Common Thai Name	Part used (Life form and Status)	Preparation/ Applications (Methods)	Health Disorders (Cook, 1995)	Symptoms and Ailments/ (Cook, 1995)	Source of Medicinal plant (threatened plants status)	UV	FIV
Acanthaceae	*Andrographis paniculata* (Burm.f.) Wall. ex Nees (AJPAC-026)	Fa thalai chon	Leaf/Whole (H)	Decoction (In)	Infections/ Infestations/ Respiratory System Disorders	Fever/Coughs	Home garden	0.33	67
	Barleria prionitis L. (AJPAC-091)	Ang kap nu	Leaf/Root (ExS)	Squeezing and Drinking (In)/Decoction (In)	Neoplasms/ Digestive System Disorders	Cancer/ Flatulence	Home garden	0.2	
	Clinacanthus nutans (Burm.f.) Lindau (AJPAC-039)	Pha ya plong thong	Leaf/Root/Whole/ Bark (HC)	Decoction (In)	Infections/ Infestations /Nutritional Disorders/ Skin/Subcutaneous Cellular Tissue Disorders	Fever/Tonic /Shingles	Home garden (R/+)	0.53	
	Justicia gendarussa Burm.f. (AJPAC-128)	Kraduk kai dam/ phra mon	Whole (ExUS)	Decoction (In)	Nutritional Disorders	Tonic in Women	National Park / Home garden	0.06	
	Phlogacanthus pulcherrimus T. Anderson. (AJPAC-116)	Di pla kang	Stem (H)	Decoction (In)	Infections/ Infestations	Fever	Community forest	0.06	
	Thunbergia grandiflora Roxb. (AJPAC-023)	Soi in tha nin	Stem (C)	Ears Drops (Ext)	Sensory System Disorders	Ear inflammation	National Park / Home garden	0.06	

Appendix S1. (Continued)

Family	Scientific names (Vourcher no.)	Common Thai Name	Part used (Life form and Status)	Preparation/ Applications (Methods)	Health Disorders (Cook, 1995)	Symptoms and Ailments/ (Cook, 1995)	Source of Medicinal plant (threatened plants status)	UV	FIV
Acanthaceae	*Thunbergia laurifolia* Lindl. (AJPAC-021)	Rang chuet	Leaf/Root (C)	Decoction (In)	Poisonings/ Digestive System Disorders/ Infections/ Infestations	Detoxicant/ Diarrhea/Fever	Community forest	0.2	
Acoraceae	*Acorus calamus* L. (AJPAC-007)	Wan nam	Rhizome/Leaf (H)	Squeezing and Drinking (In)	Skin/Subcutaneous Cellular Tissue Disorders	Allergy	Home garden	0.13	20
Amaryllidaceae	*Crinum asiaticum* L. (AJPAC-215)	Phlap phlueng	Leaf (H)	Compress (Ext)	Abnormalities	Swelling	Home garden	0.06	6
Anacardiaceae	*Anacardium occidentale* L. (AJPAC-027)	Ma muang him ma phan	Fruit (ExST)	Eat as fresh (Fd)	Injuries	Bruised	Community forest	0.13	27
	Spondias pinnata (L.f.) Kurz (AJPAC-129)	Ma kok	Bark/Leaf/Fruit/ Root/Woody (T)	Decoction (In)/ Eat as fresh (Fd)	Nutritional Disorders/Infections / Infestations /Digestive System Disorders	Tonic/Fever /Diarrhea	Community forest	0.20	
Annonaceae	*Artabotrys harmandii* Finet & Gagnep. (AJPAC-244)	Nom ngua	Whole (C)	Decoction (In)	Sensory System Disorders	Giddy	National park	0.13	13
	Goniothalamus laoticus (Finet & Gagnep.) (AJPAC-256)	Khow Lam dong	Root (S)	Grinding and Drink (In)	Poisoning	Intoxication	Community forest	0.06	
	Sphaerocoryne lefevrei (Baill.) D.M.Johnson & N.A.Murray (AJPAC-206)	Lam duan	Flower (Dry) /Woody (T)	Decoction (In)	Nutritional Disorders/ Pregnancy/Birth/ Puerperium Disorders	Tonic /Lactation	Community forest	0.13	
	Uvaria fauveliana (Finet & Gagnep.) Pierre ex Ast (AJPAC-145)	Ngo phuang phon klom	Root (C)	Decoction (In)	Nutritional Disorders	Tonic	Community forest	0.06	

Appendix S1. (Continued)

Family	Scientific names (Vourcher no.)	Common Thai Name	Part used (Life form and Status)	Preparation/ Applications (Methods)	Health Disorders (Cook, 1995)	Symptoms and Ailments/ (Cook, 1995)	Source of Medicinal plant (threatened plants status)	UV	FIV
Annonaceae	*Uvaria siamensis* (Scheff.) L.L.Zhou, Y.C.F.Su & R.M.K.Saunders (AJPAC-210)	Nom maeo	Root/Whole (S)	Decoction (In)	Digestive System Disorders	Gastritis	Community forest	0.13	
Apocynaceae	*Alyxia schlechteri* H.Lév. (AJPAC-209)	Chalut cho san	Root (C)	Decoction (In)	Infections/ Infestations	Fever	Community forest	0.26	40
	Asclepias curassavica L. (AJPAC-035)	Fai duean ha	Leaf (ExH)	Soaking and Drinking (In)	Infections/ Infestations	Fever	Community forest	0.13	
	Catharanthus roseus (L.) G.Don (AJPAC-083)	Phaeng phuai farang	Stem (ExH)	Decoction (In)	Infections/ Infestations	Fever	Community forest	0.06	
	Holarrhena curtisii King & Gamble (AJPAC-133)	Phut thung	Root (S)	Decoction (In)	Respiratory System Disorders	Asthma	Community forest (E& VU)	0.2	
	Hoya kerrii Craib (AJPAC-106)	Tang	Leaf (C)	Grilling (Ext)	Sensory System Disorders	Giddy	Community forest	0.06	
	Ichnocarpus frutescens (L.) W.T.Aiton (AJPAC-134)	Khruea pla song daeng	Root (C)	Decoction (In)	Neoplasms	Cancer	Community forest	0.06	
	Raphistemma hooperianum (Blume) Decne. (AJPAC-225)	Khao san dok lek	Root (C)	Decoction (In)	Infection/Infestations/ Neoplasms	Hepatitis/Cancer Liver	Community forest	0.20	
	Streptocaulon juventas (Lour.) Merr. (AJPAC-209)	Thao prasong	Whole/Root (C)	Decoction (In)	Injuries/ Neoplasms /Nutritional Disorders	Abscess/Cancer /Tonic	Community forest	0.20	
	Tabernaemontana bufalina Lour. (AJPAC-208)	Phrik nai phran	Root (US)	Decoction (In)	Infection/ Infestations	Fever	Community forest	0.06	
	Tabernaemontana divaricata (L.) R.Br. ex Roem. & Schult. (AJPAC-085)	Phut chip	Leaf/Root (ST)	Grinding and Smear (Ext)/ Decoction (In)	Neoplasms	Cancer	Home garden	0.13	

Appendix S1. (Continued)

Family	Scientific names (Vourcher no.)	Common Thai Name	Part used (Life form and Status)	Preparation/ Applications (Methods)	Health Disorders (Cook, 1995)	Symptoms and Ailments/ (Cook, 1995)	Source of Medicinal plant (threatened plants status)	UV	FIV
Apocynaceae	*Urceola laevigata* (Juss.) D.J.Middleton & Livsh. (AJPAC-070)	Khruea khao muak	Root (C)	Decoction (In)	Digestive System Disorders	Flatulence/ Laxative	Community forest	0.13	
	Willughbeia edulis Roxb. (AJPAC-140)	Khui	Stem (C)	Decoction (In)	Circulatory System Disorders	Epistaxis	National park	0.06	
Araceae	*Scindapsus officinalis* (Roxb.) Schott (AJPAC-129)	Khruea ngu khiao	Stem (C)	Decoction (In)	Neoplasms	Cancer	National park	0.06	20
Arecaceae	*Arenga pinnata* (Wurmb) Merr. (AJPAC-228)	Tao	Leaf (P)	Decoction (In)	Genitourinary System Disorders	Kidney stone	Community forest	0.13	13
	Calamus viminalis Willd. (AJPAC-263)	Wai khom	Rhizome/Shoot (CP)	Decoction (In)	Neoplasms	Cancer	Community forest	0.13	
	Caryota mitis Lour. (AJPAC-196)	Tao rang daeng	Root (T)	Decoction (In)	Nutritional Disorders	Tonic	Community forest	0.06	
Asparagaceae	*Dracaena cochinchinensis* (Lour.) S.C.Chen (AJPAC-055)	Chan daeng	Leaf (S)	Decoction (In)	Abnormalities	Swelling	Community forest	0.06	6
Asphodelaceae	*Aloe vera* (L.) Burm.f. (AJPAC-080)	Wan hang chora khe	Leaf (ExH)	Smear (Ext)/Eat as fresh (Fd)	Injuries	Wound	Home garden	0.2	6
Asteraceae	*Ageratum conyzoides* L. (AJPAC-191)	Sap raeng sap ka	Whole (H)	Compress (Ext)	Muscular Skeletal System Disorders	Pain	National park /Community forest	0.13	40
	Artemisia vulgaris L. (AJPAC-245)	Phit sa nat	Whole (ExH)	Decoction (In)	Sensory System Disorders	Giddy	Home garden	0.06	
	Blumea balsamifera (L.) DC. (AJPAC-025)	Nat yai	Leaf (S/ST)	Compress (Ext)	Muscular Skeletal System Disorders	Muscle pain	Home garden	0.26	
	Chromolaena odorata (L.) R.M.King & H.Rob. (AJPAC-036)	Sap suea	Leaf/Root (ExH)	Grinding and Smear (Ext)/Decoction (In)	Injuries/Pregnancy/ Birth/Puerperium Disorders	Wound/Women conditions	Home garden/ Community forest/ National park	0.20	

Appendix S1. (Continued)

Family	Scientific names (Vourcher no.)	Common Thai Name	Part used (Life form and Status)	Preparation/ Applications (Methods)	Health Disorders (Cook, 1995)	Symptoms and Ailments/ (Cook, 1995)	Source of Medicinal plant (threatened plants status)	UV	FIV
Asteraceae	*Cyanthillium cinereum* (L.) H.Rob. (AJPAC-105)	Mo noi	Root/Whole/Leaf/ Flower (H)	Grinding and drink (In)/ Decoction (In)	Infections/Infestations/ Nutritional Disorders/ Digestive System Disorders	Fever/Tonic/ Gastritis	Community forest	0.2	
	Elephantopus scaber L. var. *scaber* (AJPAC-262)	Do mai ru lom	Root (H)	Decoction (In)	Nutritional Disorders	Tonic	Community forest	0.06	
	Eupatorium fortunei Turcz. (AJPAC-253)	Phia fan	Root/Stem (H)	Decoction (In)	Muscular Skeletal System Disorders	Lumbago	Community forest	0.06	
	Mikania cordata (Burm.f.) B.L.Rob. (AJPAC-110)	Khi lek yan	Root/Whole (C)	Decoction (In)	Pregnancy/Birth/ Puerperium Disorders	Women conditions	Community forest	0.2	
Balsaminaceae	*Impatiens balsamina* L. (AJPAC-202)	Thian dok	Leaf/Flower/Seed/ Whole (ExH)	Decoction (In)	Infections/ Infestations/ Skin/Subcutaneous Cellular Tissue Disorders	Fever/Shingles	Home garden	0.13	20
Bignoniaceae	*Dolichandrone serrulata* (Wall. ex DC.) Seem. (AJPAC-233)	Khae khao	Stem (T)	Decoction (In)	Digestive System Disorders	Gastritis	National park	0.06	13
Bromeliaceae	*Ananas comosus* (L.) Merr. (AJPAC-032)	Sap pa rot	Leaf (ExH)	Decoction (In)	Digestive System Disorders	Gallstones	Home garden	0.13	6
Capparaceae	*Capparis flavicans* Kurz (AJPAC-213)	Kra chik	Stem/Leaf (S)	Decoction (In)/Bolus (In)	Nutritional Disorders/ Digestive System Disorders	Tonic /Gastritis	National park	0.2	13
Celastraceae	*Salacia chinensis* L. (AJPAC-120)	Kam phaeng chet chan	Root/Whole (ScanS)	Decoction (In)	Digestive System Disorders	Diarrhea/ Gastritis	Community forest	0.13	20
Chrysobalanaceae	*Parinari anamensis* Hance (AJPAC-119)	Ma phok	Woody/Bark (T)	Decoction (In)	Nutritional Disorders	Tonic heart	Community forest	0.13	13

Appendix S1. (Continued)

Family	Scientific names (Vourcher no.)	Common Thai Name	Part used (Life form and Status)	Preparation/ Applications (Methods)	Health Disorders (Cook, 1995)	Symptoms and Ailments/ (Cook, 1995)	Source of Medicinal plant (threatened plants status)	UV	FIV
Combretaceae	*Getonia floribunda* Roxb. (AJPAC-132)	Ting tang	Stem/Woody (CS)	Squeezing and Eye drops (Ext) /Decoction (In)	Sensory System Disorders/ Infections/Infestations/ Digestive System Disorders/	Eye vision /Fever/Diarrhea	National park /Community forest	0.2	26
	Terminalia bellirica (Gaertn.) Roxb. (AJPAC-086)	Samo phi phek	Fruit (T)	Decoction (In)/ Grinding and drink (In)	Respiratory System Disorders	Coughs	Community forest	0.2	
	Terminalia chebula Retz. (AJPAC-020)	Sa mo thai	Fruit/Seed/Woody (T)	Eat as fresh (Fd)/ Decoction (In)	Digestive System Disorders	Laxative	Community forest	0.20	
	Terminalia mucronata Craib & Hutch. (AJPAC-123)	Ma kluea lueat	Leaf/Bark (T)	Decoction (In)	Digestive System Disorders	Diarrhea/ Gastritis	Community forest	0.20	
	Terminalia nigrovenulosa Pierre (AJPAC-211)	Khi ai	Bark (T)	Chewing (In)	Inflammations	Mouth ulcers	National park	0.2	
Commelinaceae	*Tradescantia spathacea* Sw. (AJPAC-069)	Wan hoi khraeng	Leaf (ExH)	Squeezing and smear (Ex)	Skin/Subcutaneous Cellular Tissue Disorders	Dermatosis	Home garden	0.33	6
Connaraceae	*Connarus semidecandrus* Jack (AJPAC-103)	Thop thaep khruea	Stem/Root/Leaf (C)	Decoction (In)	Infections/Infestations/Digestive System Disorders	Fever/Diarrhea/ Gastritis	Community forest /National park	0.2	20
Costaceae	*Hellenia speciosa* (J.Koenig) S.R.Dutta (AJPAC-204)	Ueang mai na	Root/Rhizome (H)	Decoction (In)/Eat as fresh (Fd)	Respiratory System Disorders/ Infections/Infestations	Coughs/ Anthelminthic	Buy	0.13	13
Cucurbitaceae	*Coccinia grandis* (L.) Voigt (AJPAC-045)	Phak tam lueng	Leaf (HC)	Squeezing (In)	Infections/Infestations	Fever	Home garden	0.06	13

Appendix S1. (Continued)

Family	Scientific names (Vourcher no.)	Common Thai Name	Part used (Life form and Status)	Preparation/ Applications (Methods)	Health Disorders (Cook, 1995)	Symptoms and Ailments/ (Cook, 1995)	Source of Medicinal plant (threatened plants status)	UV	FIV
Cyperaceae	*Scleria levis* Retz. (AJPAC-067)	Ya sam khom	Whole (H)	Decoction (In)	Neoplasms/ Genitourinary System Disorders	Cancer/ Leukorrhea	National park	0.13	13
Dioscoreaceae	*Dioscorea hispida* Dennst. (AJPAC-216)	Kloi	Tuber (HC)	Dry and Decoction (In)	Neoplasms	Cancer	National park	0.13	13
Dipterocarpaceae	*Hopea odorata* Roxb. (AJPAC-200)	Ta khian thong	Bark (T)	Decoction (In)/ Grinding and Smear (Ext)	Muscular Skeletal System Disorders/	Muscle Pain	Home garden	0.13	20
Elaeagnaceae	*Elaeagnus latifolia* L. (AJPAC-129)	Ma lot	Root (C)	Decoction (In)	Nutritional Disorders/	Tonic	National park	0.06	13
Erythroxylaceae	*Erythroxylum cuneatum* (Miq.) Kurz (AJPAC-130)	Krai thong	Root/Leaf (ST)	Decoction (In)	Nutritional Disorders/ Pregnancy/Birth/ Puerperium Disorders	Tonic /Lactation	Community forest	0.13	13
Euphorbiaceae	*Aporosa villosa* (Lindl.) Baill. (AJPAC-193)	Lot	Bark (ST)	Decoction (In)	Digestive System Disorders/ Pregnancy/Birth/ Puerperium Disorders	Flatulence/ Haemorrhage of pregnancy	Community forest	0.13	66
	Croton crassifolius Geiseler (AJPAC-232)	Phang khi	Root/Whole (US)	Decoction (In)/ Compress (Ext)/ Grinding and Smear (Ext)	Digestive System Disorders	Flatulence	Community forest /Home garden	0.2	
	Croton persimilis Müll.Arg. (AJPAC-249)	Plao yai	Leaf/Whole/Bark/ Fruit (ST)	Compress (Ext)/Decoction (In)/ Eat as fresh (Fd)	Muscular Skeletal System Disorders/ Endocrine System Disorders	Muscle pain /Diabetes	Community forest	0.26	

Appendix S1. (Continued)

Family	Scientific names (Vourcher no.)	Common Thai Name	Part used (Life form and Status)	Preparation/ Applications (Methods)	Health Disorders (Cook, 1995)	Symptoms and Ailments/ (Cook, 1995)	Source of Medicinal plant (threatened plants status)	UV	FIV
Euphorbiaceae	*Euphorbia tirucalli* L. (AJPAC-234)	Phaya rai bai	Root/Gum (ExST)	Grinding and Smear (Ext)/Smear (Ext)	Poisonings	Detoxicant	Home garden	0.13	66
	Jatropha curcas L. (AJPAC-001)	Sabu dam	Whole/Leaf/Gum (ExS)	Boil and bath (Ext)/ Smear (Ext)/Squeezing and drink (In)	Sensory System Disorders	Giddy	Home garden	0.13	
	Sampantaea amentiflora (Airy Shaw) Airy Shaw (AJPAC-066)	Sam phan ta	Leaf (S)	Decoction (In)/Inhale (In)	Sensory System Disorders	Giddy	National park	0.13	
	Suregada multiflora (A.Juss.) Baill. (AJPAC-144)	Khan thong phayabat	Root (ST)	Decoction (In)	Respiratory System Disorders	Coughs	Community forest	0.13	
	Trigonostemon reidioides (Kurz) Craib (AJPAC-136)	Lot thanong	Root (US)	Grinding and smear (Ext)	Digestive System Disorders /Poisonings	Toothache/ Detoxicant	Community forest /National park	0.13	
Fabaceae	*Albizia myriophylla* Benth. (AJPAC-194)	Cha em pa	Leaf/Root/Stem (C)	Eat as fresh (In)/ Decoction (In)	Infection/Infestations/Pregnancy/Birth/ Puerperium Disorders	Malaria/Lactation	Community forest /Home garden	0.46	93
	Barnebydendron riedelii (Tul.) J.H.Kirkbr. (AJPAC-212)	Pradu daeng	Flower (T)	Pickled liquor (In)	Nutritional Disorders	Tonic	National park	0.13	
	Cassia fistula L. (AJPAC-095)	Ratcha phruek	Whole (T)	Decoction (In)	Digestive System Disorders	Gastritis	Community forest	0.06	

Appendix S1. (Continued)

Family	Scientific names (Vourcher no.)	Common Thai Name	Part used (Life form and Status)	Preparation/ Applications (Methods)	Health Disorders (Cook, 1995)	Symptoms and Ailments/ (Cook, 1995)	Source of Medicinal plant (threatened plants status)	UV	FIV
Fabaceae	*Cheniella lakhonensis* (Gagnep.) R.Clark & Mackinder (AJPAC-248)	Som siao thao	Leaf/Bark (C)	Decoction (In)	Pregnancy/Birth/ Puerperium Disorders /Respiratory System Disorders	Hemorrhage of pregnancy/ Coughs	Community forest	0.13	
	Clitoria ternatea L. (AJPAC-042)	Anchan	Root (ExC)	Grinding and dring (In)	Genitourinary System Disorders	Diuretic	Home garden	0.06	
	Dalbergia pinnata (Lour) Prain (AJPAC-197)	Ma kham khruea	Root (C)	Decoction (In)	Nutritional Disorders	Tonic	Community forest	0.06	
	Dalbergia velutina Benth. (AJPAC-250)	Khruea khang khwai	Bark (C)	Eat as fresh (Fd)	Pregnancy/Birth/ Puerperium Disorders/ Injuries	Jaundice/Wound	Community forest	0.13	
	Droogmansia godefroyana (Kuntze) Schindl. (AJPAC-251)	Chai hin	Woody (S)	Decoction (In)	Nutritional Disorders	Tonic	Community forest	0.06	
	Erythrina subumbrans (Hassk.) Merr. (AJPAC-261)	Thong lang pa	Root (T)	Decoction (In)	Muscular Skeletal System Disorders	Pain	National park	0.06	
	Lasiobema penicillilobum (Pierre ex Gagnep.) A Schmitz (AJPAC-220)	Siao daeng	Stem (C)	Decoction (In)	Nutritional Disorders/ Muscular Skeletal System Disorders/	Tonic /Pain	National park	0.46	
	Lasiobema pulla (Craib) A.Schmitz (AJPAC-237)	Salaeng phan thao	Bark (C)	Soaking and Drink (In)	Circulatory System Disorders	Hemorrhoids	National park	0.06	

Appendix S1. (Continued)

Family	Scientific names (Vourcher no.)	Common Thai Name	Part used (Life form and Status)	Preparation/ Applications (Methods)	Health Disorders (Cook, 1995)	Symptoms and Ailments/ (Cook, 1995)	Source of Medicinal plant (threatened plants status)	UV	FIV
Fabaceae	*Lysiphyllum strychnifolium* (Craib) A.Schmitz (AJPAC-221)	Khayan	Root/Leaf/ Rhizome/ Stem (C)	Decoction (In)	Endocrine System Disorders/ /Nutritional Disorders/ Circulatory System Disorders / Poisonings/	Diabetes/ Cardiopathy/Tonic/Hypertension/ Detoxicant	Community forest/ National park (E&NT)	0.46	
	Mimosa pudica L. (AJPAC-052)	Mai ya rap	Root/Leaf/Stem/ Whole (H)	Decoction (In)	Infections/Infestations / Digestive System Disorders	Fever/Gall stone	Home garden	0.26	
	Phanera curtisii (Prain) Bandyop. & Ghoshal (AJPAC-241)	Khruea khao kaep	Root (CS)	Decoction (In)	Pregnancy/Birth/Puerperium Disorders	Lactation	Community forest	0.06	
	Phyllocarpus septentrionalis J.D.Sm.	Pra du dang	Flower (T)	Fermented (In)	Nutritional Disorders/	Tonic	National park /Home garden	0.13	
	Phyllodium longipes (Craib) Schindl. (AJPAC-224)	Klet pla	Root (US)	Decoction (In)	Genitourinary System Disorders	Leukorrhoea	National park	0.06	
	Piliostigma malabaricum (Roxb.) Benth. (AJPAC-242)	Siao yai	Leaf/Bark (ST)	Decoction (In)	Circulatory System Disorders /Respiratory System Disorders	Haemagogue / Coughs	National park	0.13	
	Pterocarpus macrocarpus Kurz (AJPAC-127)	Pradu pa	Root/Woody/Bark (T)	Decoction (In)	Infections/ Infestations/Digestive System Disorders/ Muscular Skeletal System Disorders/	Fever/ Diarrhea/ Pain	Community forest	0.26	
	Pueraria mirifica Airy Shaw & Suvat. (AJPAC-141)	Kwao khruea	Tuber (C)	Decoction (In)	Nutritional Disorders/ Neoplasms	Tonic/Cancer	Community forest	0.2	

Appendix S1. (Continued)

Family	Scientific names (Vourcher no.)	Common Thai Name	Part used (Life form and Status)	Preparation/ Applications (Methods)	Health Disorders (Cook, 1995)	Symptoms and Ailments/ (Cook, 1995)	Source of Medicinal plant (threatened plants status)	UV	FIV
Fabaceae	*Senegalia pennata* (L.) Maslin subsp. *insuavis* (Lace) Maslin, Seigler & Ebinger (AJPAC-068)	Cha om	Root/Shoot (SC)	Decoction (In)/ Eat as fresh (Fd)	Muscular Skeletal System Disorders	Pain	Home garden	0.13	
	Senegalia rugata (Lam.) Britton & Rose (AJPAC-087)	Som poi	Root/Leaf (SC)	Eat as fresh (Fd)/ Decoction (In)	Digestive System Disorders	Flatulence	Home garden	0.2	
	Sesbania grandiflora (L.) Poir. (AJPAC-128)	Khae ban	Root/Woody/Bark (ExST)	Decoction (In)/ Soaking and Drink (In)	Infections/ Infestations	Fever	Home garden	0.2	
	Sindora siamensis Teijsm. ex Miq. (AJPAC-124)	Ma kha tae	Fruit/Woody (T)	Decoction (In)	Muscular Skeletal System Disorders/	Pain	Community forest /National park	0.13	
	Tadehagi triquetrum (L.) H.Ohashi (AJPAC-131)	Khao mao nok	Root (H)	Decoction (In)	Genitourinary System Disorders	Leukorrhea	Community forest	0.06	
	Tamarindus indica L. (AJPAC-015)	Ma kham	Root/Leaf (ExT)	Decoction (In)/ Compress (Ext)	Inflammations/ Infections/ Infestations/	Mouth ulcers/ Chicken pok/ Fever	Home garden	0.26	
	Uraria crinita (L.) Desv. ex DC. (AJPAC-14)	Hang ma chok	Root (US)	Decoction (In)	Infections/Infestatio ns	Anthelminthic	National park	0.13	
	Xylia xylocarpa (Roxb.) W.Theob. (AJPAC-138)	Daeng	Woody/Bark/Root /Leaf (T)	Decoction (In)/Boil and bath (Ext)/Compres s (Ext)/Eat as fresh (Fd)	Muscular Skeletal System Disorders/ /Infections/ Infestations/ Digestive System Disorders	Pain/Fever/ Diarrhea/ Flatulence	Community forest/ National park	0.33	
Fagaceae	*Castanopsis piriformis* Hickel & A.Camus (AJPAC-214)	Ko hin	Root (T)	Decoction (In)	Injuries	Wound	Community forest	0.06	15

Appendix S1. (Continued)

Family	Scientific names (Vourcher no.)	Common Thai Name	Part used (Life form and Status)	Preparation/ Applications (Methods)	Health Disorders (Cook, 1995)	Symptoms and Ailments/ (Cook, 1995)	Source of Medicinal plant (threatened plants status)	UV	FIV
Fagaceae	*Quercus helferiana* A.DC. (AJPAC-128)	Ko khi mu	Stem (T)	Decoction (In)	Inflammations	Mouth ulcers	National park	0.06	
Gnetaceae	*Gnetum montanum* Markgr. (AJPAC-255)	Mueai	Stem (C)	Decoction (In)	Nutritional Disorders	Tonic	Community forest	0.20	20
Iridaceae	*Iris domestica* (L.) Goldblatt & Mabb. (AJPAC-236)	Wan hang chang	Rhizome (H)	Decoction (In)	Infections/ Infestations	Fever	Home garden	0.06	20
	Sisyrinchium palmifolium L. (AJPAC-207)	Wan hom daeng	Tuber (H)	Decoction (In)	Sensory System Disorders	Giddy	Home garden	0.06	
Irvingiaceae	*Irvingia malayana* Oliv. ex A.W.Benn. (AJPAC-258)	Krabok	Woody (T)	Decoction (In)	Infections/ Infestations/Respira tory System Disorders	Fever/Coughs	Community forest	0.26	20
Lamiaceae	*Clerodendrum schmidtii* C.B.Clarke (AJPAC-099)	Phut ra cha	Leaf (S)	Decoction (In)	Pregnancy Birth/Puerperium Disorders	Abortions	National park	0.06	33
	Coleus amboinicus Lour. (AJPAC-050)	Niam hu suea	Leaf (H)	Inhale (In)/Smear (Ext)	Infections/Infestatio ns /Nutritional Disorders	Fever/ Tonic	Home garden	0.13	
	Mesosphaerum suaveolens (L.) Kuntze (AJPAC-139)	Maeng lak kha	Leaf (H)	Decoction (In)	Nervous System Disorders	Migraines	Community forest	0.06	
	Ocimum × africanum Lour. (AJPAC-240)	Maeng lak	Root (ExH)	Decoction (In)	Respiratory System Disorders	Pneumonia	Home garden	0.06	
	Orthosiphon aristatus (Blume) Miq. (AJPAC-259)	Ya nuat maeo	Leaf/Stem (H)	Decoction (In)	Muscular Skeletal System Disorders/ Digestive System Disorders	Muscle pain / Gallstones	Community forest /Home garden	0.2	
	Vitex peduncularis Wall. ex Schauer (AJPAC-137)	Ka sam pik	Root (T)	Decoction (In)	Nutritional Disorders	Tonic	Community forest	0.06	
	Vitex pinnata L. (AJPAC-084)	Tin nok	Woody/Root (T)	Decoction (In)	Digestive System Disorders	Diarrhea	National park	0.13	

Appendix S1. (Continued)

Family	Scientific names (Vourcher no.)	Common Thai Name	Part used (Life form and Status)	Preparation/ Applications (Methods)	Health Disorders (Cook, 1995)	Symptoms and Ailments/ (Cook, 1995)	Source of Medicinal plant (threatened plants status)	UV	FIV
Lauraceae	*Cinnamomum parthenoxylon* (Jack) Meisn. (AJPAC-096)	Thep tharo	Root/Bark/Woody (T)	Decoction (In)	Infections/ Infestations	Fever	Community forest/Buy	0.13	33
	Litsea cubeba (Lour.) Pers. (AJPAC-127)	Ta khrai ton	Root/Fruit (ST)	Grinding and drink (In)/ Eat as fresh (Fd)	Muscular Skeletal System Disorders/ /Digestive System Disorders	Pain/Flatulence	Community forest	0.13	
Lecythidaceae	*Barringtonia racemosa* (L.) Spreng. (AJPAC-195)	Chik suan	Root/Woody (ST)	Decoction (In)	Infections/ Infestations /Digestive System Disorders	Fever/Diarrhea	Community forest	0.2	27
	Careya arborea Roxb. (AJPAC-093)	Kra don	Bark/Rhizome (T)	Decoction (In)/ Compress (Ext)	Digestive System Disorders	Gastritis	Community forest	0.20	
Loganiaceae	*Strychnos axillaris* Colebr. (AJPAC-121)	Khwak kai	Root/Stem/Leaf/ Woody/Fruit/Wh ole (C)	Grinding and drink (In)/ Decoction (In)	Infections/ Infestations /Nutritional Disorders/	Fever/Tonic	Community forest	0.4	27
	Strychnos nux-blanda A.W. Hill. (AJPAC-122)	Tumka khao	Root (ST)	Decoction (In)	Muscular Skeletal System Disorders/ /Infections/ Infestations	Pain/Fever	Community forest	0.13	
	Strychnos nux-vomica L. (AJPAC-123)	Salaeng chai	Woody (ST)	Decoction (In)	Infections/ Infestations	Fever	Community forest	0.06	
Lythraceae	*Punica granatum* L. (AJPAC-004)	Thap thim	Bark/Fruit/Root (ExS)	Grinding and drink (In)/Decoction (In)/ Eat as fresh (Fd)	Muscular Skeletal System Disorders/	Pain	Home garden	0.2	13

Appendix S1. (Continued)

Family	Scientific names (Vourcher no.)	Common Thai Name	Part used (Life form and Status)	Preparation/ Applications (Methods)	Health Disorders (Cook, 1995)	Symptoms and Ailments/ (Cook, 1995)	Source of Medicinal plant (threatened plants status)	UV	FIV
Malvaceae	*Bombax anceps* Pierre (AJPAC-024)	Ngio pa	Stem (T)	Bolus (In)	Pregnancy/Birth/Puerperium Disorders	Lactation	National park	0.06	20
	Gossypium herbaceum L. (AJPAC-105)	Fai	Leaf/Seed (ExS)	Decoction (In)	Infections/ Infestations /Digestive System Disorders	Fever/ Gallstones	Home garden	0.13	
	Sterculia guttata Roxb. (AJPAC-121)	Po daeng	Root (S)	Decoction (In)	Circulatory System Disorders	Hemorrhoids	Community forest	0.06	
Melastomataceae	*Memecylon edule* Roxb. (AJPAC-109)	Phlong mueat	Woody/Root (S/ST)	Grinding and drinking (In) /Decoction (In)	Infections/ Infestations	Fever/ Tuberculosis	Community forest	0.13	16
Meliaceae	*Walsura trichostemon* Miq. (AJPAC-125)	Kat lin	Woody (T)	Decoction (In)	Muscular Skeletal System Disorders	Lumbago	National park	0.06	13
Menispermaceae	*Cissampelos pareira* L. (AJPAC-231)	Krung kha mao	Leaf (C)	Decoction (In)	Inflammations	Mouth ulcers	National park	0.13	53
	Stephania pierrei Diels (AJPAC-013)	Sabu lueat	Tuber (C)	Grinding and drinking (In)	Neoplasms	Cancer	Home garden	0.2	
	Stephania venosa (Blume) Spreng. (AJPAC-089)	Kra thom lueat	Tuber (C)	Decoction (In)	Pregnancy/Birth/Puerperium Disorders	Haemorrhage of pregnancy	Community forest	0.06	
	Tiliacora triandra (Colebr.) Diels (AJPAC-134)	Thao ya nang	Leaf/Root/Stem (C)	Squeezing and drinking (In) /Decoction (In)	Infections/ Infestations / Endocrine System Disorders/ Circulatory System Disorders	Fever/Diabetes/ Hemorrhoids	Home garden /Community forest	0.33	

Appendix S1. (Continued)

Family	Scientific names (Vourcher no.)	Common Thai Name	Part used (Life form and Status)	Preparation/ Applications (Methods)	Health Disorders (Cook, 1995)	Symptoms and Ailments/ (Cook, 1995)	Source of Medicinal plant (threatened plants status)	UV	FIV
Menispermaceae	*Tinospora crispa* (L.) Hook.f. & Thomson (AJPAC-124)	Bora phet	Leaf/Root/Stem (C)	Eat as fresh (Fd)/Decoction (In)	Skin/Subcutaneous cellular tissue disorders/Infections / Infestations	Allergy/Fever	Home garden	0.26	
Moraceae	*Artocarpus lacucha* Buch.-Ham. (AJPAC-029)	Ma Hat	Woody/Bark (T)	Decoction (In)	Infections/Infestations	Anthelminthic	Community forest	0.33	60
	Ficus hirta Vahl (AJPAC-198)	Ma duea hom	Root/Fruit/Stem (S/ST)	Decoction (In)/ Eat as fresh (Fd)	Injuries /Neoplasms /Nutritional Disorders	Abscess/Cancer/ Tonic heart	Community forest	0.2	
	Ficus foveolata Wall. (AJPAC-131)	Ma kra tub rong	Stem (SC)	Decoction (In)	Nutritional Disorders/	Tonic	Community forest	0.06	
	Ficus hispida (AJPAC-235)	Ma duea plong	Root/Fruit (T)	Decoction (In)/ Eat as fresh (Fd)	Injuries /Neoplasms	Abscess/Cancer	Community forest	0.13	
	Streblus asper Lour. (AJPAC-130)	Khoi	Woody/Root/Bark (T)	Decoction (In)	Nutritional Disorders/	Tonic/Tonic heart	Community forest	0.33	
Moringaceae	*Moringa oleifera* Lam. (AJPAC-111)	Ma rum	Root/Fruit (ST)	Decoction (In)	Infections//Infestations	Fever	Home garden	0.13	13
Musaceae	*Musa* (ABB) 'Kluay Tip' (AJPAC-061)	Kluy	Leaf (Leaf sheet) (ExH)	Boil and bath (Ext)	Infection /Infestations	Chicken pok	Home garden	0.06	13
Myrtaceae	*Psidium guajava* L. (AJPAC-003)	Farang	Leaf (ExST)	Decoction (In)	Digestive System Disorders	Diarrhea	Home garden	0.13	40
	Rhodomyrtus tomentosa (Aiton) Hassk. (AJPAC-118)	Phruat	Root (S)	Decoction (In)	Digestive System Disorders	Diarrhea	Community forest	0.2	
	Syzygium antisepticum (Blume) Merr. & L.M.Perry (AJPAC-113)	Samet chun	Woody (ST)	Decoction (In)	Pregnancy/Birth/Puerperium Disorders	Jaundice	National park	0.13	
Nyctaginaceae	*Mirabilis jalapa* L. (AJPAC-049)	Ban yen	Root (H)	Decoction (In)	Infections/ Infestations	Cystitis	Community forest	0.26	13

Appendix S1. (Continued)

Family	Scientific names (Vourcher no.)	Common Thai Name	Part used (Life form and Status)	Preparation/ Applications (Methods)	Health Disorders (Cook, 1995)	Symptoms and Ailments/ (Cook, 1995)	Source of Medicinal plant (threatened plants status)	UV	FIV
Ochnaceae	*Campylospermum serratum* (Gaertn.) Bittrich & M.C.E.Amaral (AJPAC-247)	Chang nom	Woody (S)	Decoction (In)	Nutritional Disorders	Tonic	Community forest	0.06	33
	Ochna integerrima (Lour.) Merr. (AJPAC-125)	Chang nao	Stem/Bark/Fruit/ Root (S/ST)	Decoction (In)	Digestive System Disorders	Gastritis/ Flatulence	Community forest	0.33	
Olacaceae	*Olax psittacorum* (Lam.) Vahl (AJPAC-126)	Nam chai khrai	Leaf (C)	Decoction (In)	Infections/Infestations	Cold	Community forest	0.26	13
Opiliaceae	*Melientha suavis* Pierre (AJPAC-138)	Phak wan	Whole (S/ST)	Decoction (In)	Nutritional Disorders	Tonic	National park	0.13	13
Oxalidaceae	*Biophytum sensitivum* (L.) DC. (AJPAC-229)	Mai ya rap	Whole (H)	Decoction (In)	Nutritional Disorders	Tonic	National park	0.06	13
Phyllanthaceae	*Antidesma puncticulatum* Miq. (AJPAC-090)	Mao luang	Fruit/Whole/Root (ST)	Decoction (In) / Eat as fresh (Fd)	Digestive System Disorders/ Pregnancy/Birth/Puerperium Disorders	Flatulence/ Haemorrhage of pregnancy	Community forest	0.20	40
	Baccaurea ramiflora Lour. (AJPAC-030)	Ma fai	Root/Woody/fruit (T)	Decoction (In)/ Eat as fresh (Fd)	Infections/ Infestations /Digestive System Disorders	Fever/Laxative	Community forest	0.13	
	Phyllanthus acidus (L.) Skeels (AJPAC-062)	Ma yom	Woody (ExST)	Soaking and Drink (In)	Muscular Skeletal System Disorders/	Pain	Home garden	0.06	
	Phyllanthus amarus Schumach. & Thonn. (AJPAC-065)	Luk tai bai	Root/Leaf (H)	Decoction (In)	Infections/ Infestations	Fever	Home garden	0.13	
	Phyllanthus emblica L. (AJPAC-064)	Ma kham pom	Root/Fruit (T)	Eat as fresh (In)/Decoction (In)	Nutritional Disorders/Respiratory System Disorders	Tonic/ Coughs	Community forest	0.13	

Appendix S1. (Continued)

Family	Scientific names (Vourcher no.)	Common Thai Name	Part used (Life form and Status)	Preparation/ Applications (Methods)	Health Disorders (Cook, 1995)	Symptoms and Ailments/ (Cook, 1995)	Source of Medicinal plant (threatened plants status)	UV	FIV
Piperaceae	*Piper nigrum* L. (AJPAC-010)	Phrik thai	Stem/Leaf/Root/Seed (ExC)	Decoction (In)/ Eat as fresh (Fd)	Digestive System Disorders/ Circulatory System Disorders/ Endocrine System Disorders	Flatulence/ Hypertension / Diabetes	Home garden	0.40	40
	Piper ribesioides Wall. (AJPAC-124)	Ta khan lek	Stem/Leaf (C)	Decoction (In)	Sensory System Disorders	Giddy	Community forest	0.06	
	Piper sarmentosum Roxb. (AJPAC-012)	Cha phlu	Whole (CrH)	Decoction (In)	Endocrine System Disorders	Diabetes	Home garden	0.06	
Plantaginaceae	*Limnophila aromatica* (Lam.) Merr. (AJPAC-137)	Phak kha yaeng	Whole (H)	Eat as fresh (Fd)	Sensory System Disorders	Giddy	Community forest	0.06	6
Plumbaginaceae	*Plumbago indica* L. (AJPAC-014)	Chetta mun phloeng daeng	Root (US)	Decoction (In)	Pregnancy/Birth/Puerperium Disorders	Haemorrhage of pregnancy	Home garden	0.20	20
Poaceae	*Centotheca lappacea* (L.) Desv. (AJPAC-230)	Ya niao ma	Whole (G)	Decoction (In)	Infections/ Infestations /Skin/Subcutaneous Cellular Tissue Disorders	Fever/Shingles	Community forest	0.2	67
	Cymbopogon citratus (DC.) Stapf (AJPAC-053)	Ta khrai	Root/Leaf (ExG)	Eat as fresh (Fd)/Decoction (In)/ Compress (Ext)	Infections/ Infestations /Digestive System Disorders/ Muscular Skeletal System Disorders/	Fever/Diarrhea/ Pain	Home garden	0.33	
	Cynodon dactylon (L.) Pers. (AJPAC-232)	Ya phraek	Whole (ExG)	Decoction (In)	Sensory System Disorders	Giddy	Community forest	0.06	
	Imperata cylindrica (L.) Raeusch. (AJPAC-219)	Ya kha	Stem/Whole (Dry) (G)	Decoction (In)	Genitourinary System Disorders	Kidney stones	Community forest	0.06	

Appendix S1. (Continued)

Family	Scientific names (Vourcher no.)	Common Thai Name	Part used (Life form and Status)	Preparation/ Applications (Methods)	Health Disorders (Cook, 1995)	Symptoms and Ailments/ (Cook, 1995)	Source of Medicinal plant (threatened plants status)	UV	FIV
Poaceae	*Saccharum officinarum* L. (AJPAC-005)	Oi dang	Whole (G)	Decoction (In)	Pregnancy/Birth/Puerperium Disorders	Lactation	Home garden	0.06	
	Saccharum × *sinense* Roxb. (AJPAC-006)	Oi dum	Root/Whole (ExG)	Decoction (In)	Digestive System Disorders/ Circulatory System Disorders	Gall stone/ Hemorrhoids/ Gastritis	Home garden	0.2	
	Vietnamosasa pusilla (A.Chev. & A.Camus) T.Q.Nguyen (AJPAC-141)	Phek	Rhizome (B)	Decoction (In)	Infections/ Infestations/ Nervous System disorders	Fever/ Migraines	National park	0.13	
Polygonaceae	*Muehlenbeckia platyclada* (F.Muell.) Meisn. (AJPAC-222)	Ta khap hin	Leaf/Whole (ExS)	Compress (Ext)/Squeezing and smear (Ext)	Endocrine System Disorders /Circulatory System Disorders	Diabetes/ Hypertension	Home garden	0.13	20
Polypodiaceae	*Drynaria quercifolia* (L.) J.Sm. (AJPAC-217)	Kratae tai mai	Rhizome (EF)	Decoction (In)	Digestive System Disorders	Gallstones	National park	0.2	13
Pontederiaceae	*Pontederia vaginalis* Burm.f. (AJPAC-117)	Phak khiat	Leaf (ExAqH)	Eat as fresh (Fd)	Infections/ /Infestations/ Skin/Subcutaneous Cellular Tissue Disorders	Fever/Shingles	Home garden	0.13	6
Primulaceae	*Ardisia* sp. (AJPAC-211)	Kei tai	Stem/Root/Leaf (S)	Decoction (In)	Digestive System Disorders	Gastritis	Community forest	0.13	6
Rhamnaceae	*Ventilago denticulata* Willd. (AJPAC-142)	Rang daeng	Root (C)	Decoction (In)	Pregnancy/Birth/ Puerperium Disorders	Lactation	Community forest	0.06	26
	Ziziphus oenopolia (L.) Mill. (AJPAC-001)	Lep yiao	Root (C)	Decoction (In)	Endocrine System Disorders	Diabetes	Community forest	0.06	

Appendix S1. (Continued)

Family	Scientific names (Vourcher no.)	Common Thai Name	Part used (Life form and Status)	Preparation/ Applications (Methods)	Health Disorders (Cook, 1995)	Symptoms and Ailments/ (Cook, 1995)	Source of Medicinal plant (threatened plants status)	UV	FIV
Rubiaceae	*Canthium berberidifolium* E.T.Geddes (AJPAC-092)	Ngiang duk	Woody (S)	Decoction (In)	Muscular Skeletal System Disorders	Pain	National park	0.06	26
	Catunaregam tomentosa (Blume ex DC.) Tirveng. (AJPAC-094)	Ma khet	Whole (T)	Decoction (In)	Neoplasms / Infections/ Infestations/Genitourinary System Disorders	Liver Cancer/Fever/ Diuretic	Community forest	0.2	
Rubiaceae	*Gardenia sootepensis* Hutch. (AJPAC-199)	Kham mok luang	Root/Woody (ST)	Decoction (In)	Digestive System Disorders	Diarrhea	Community forest	0.13	
Rubiaceae	*Ixora lobbii* Van Houtte ex Bosse (AJPAC-107)	Khem daeng	Root (S)	Decoction (In)	Circulatory System Disorders/ Pregnancy/Birth/Puerperium Disorders	Hemorrhoids/ Haemorrhage of pregnancy	Community forest /Home garden	0.13	
Rubiaceae	*Ixora lucida* R.Br. ex Hook.f. (AJPAC-108)	Khem khao	Root/Stem (S)	Decoction (In)	Neoplasms/ Pregnancy/Birth/Puerperium Disorders	Cancer/ Haemorrhage of pregnancy	Community forest /Home garden	0.13	
Rubiaceae	*Mitragyna diversifolia* (Wall. ex G.Don) Havil. (AJPAC-205)	Kra thum na	Leaf (S/ST)	Eat as fresh (Fd)	Endocrine System Disorders /Circulatory System Disorders	Diabetes/ Hypertension	Community forest	0.13	
Rubiaceae	*Morinda coreia* Buch.-Ham. (AJPAC-060)	Yo pa	Root/Leaf/Woody (ST)	Decoction (In)	Digestive System Disorders/ Birth/Puerperium Disorders	Flatulence/ Jaundice	Community forest	0.2	
Rubiaceae	*Neonauclea sessilifolia* (Roxb.) Merr. (AJPAC-239)	Kra thum hu kwang	Leaf/Bark (T)	Decoction (In)	Infections/ Infestations/ Circulatory System Disorders	Fever/ Hypertension	National park	0.13	

Appendix S1. (Continued)

Family	Scientific names (Vourcher no.)	Common Thai Name	Part used (Life form and Status)	Preparation/ Applications (Methods)	Health Disorders (Cook, 1995)	Symptoms and Ailments/ (Cook, 1995)	Source of Medicinal plant (threatened plants status)	UV	FIV
Rubiaceae	*Oxyceros horridus* Lour. (AJPAC-223)	Khat khao khruea	Stem/Root (ScanS)	Decoction (In)	Genitourinary System Disorders /Neoplasms	Uterus conditions /Cancer	Community forest	0.13	
Rubiaceae	*Paederia linearis* Hook.f. (AJPAC-115)	Tot mu tot ma	Stem (C)	Decoction (In)	Digestive System Disorders	Flatulence / Diarrhea	Community forest	0.13	
Rubiaceae	*Ridsdalea wittii* (Craib) J.T.Pereira (AJPAC-243)	Mak mo	Woody (T)	Decoction (In)	Pregnancy/Birth/Puerperium Disorders	Lactation	National park	0.06	
Rutaceae	*Acronychia pedunculata* (L.) Miq. (AJPAC-071)	Ka uam	Woody (S/ST)	Decoction (In)	Digestive System Disorders/Pregnancy/Birth/Puerperium Disorders	Gastritis/Women conditions	Community forest	0.13	80
	Aegle marmelos (L.) Corrêa (AJPAC-089)	Ma tum	Root/Leaf/ Bark (T)	Decoction (In)/Squeezing (Ext)	Skin/Subcutaneous Cellular Tissue Disorders	Allergy	Community forest	0.13	
	Atalantia monophylla DC. (AJPAC-192)	Ma nao phi	Woody (ST)	Decoction (In)	Circulatory System Disorders	Hemorrhoids	National park	0.13	
	Citrus hystrix DC. (AJPAC-041)	Ma krut	Leaf/Fruit/Bark/ Root (ST)	Compress (Ext)/ Decoction (In)	Muscular Skeletal System Disorders/ /Digestive System Disorders	Pain/Flatulence	Home garden	0.13	
	Citrus aurantiifolia (Christm.) Swingle (AJPAC-043)	Ma nao	Root/Leaf/Fruit (ExST)	Decoction (In)/ Eat as fresh (Fd)	Infection/Infestations/ Neoplasms	Tuberculosis/ Cancer	Home garden	0.46	
	Clausena wallichii Oliv. (AJPAC-038)	Phia fan	Root/Leaf (S)	Decoction (In)/ Eat as fresh (Fd)	Infections//Infestations/Poisonings	Fever/ Detoxicant	Community forest	0.13	
	Feroniella lucida (Scheff.) Swingle (AJPAC-254)	Ma sang	Root (ST)	Decoction (In)	Infections/ Infestations	Fever	Home garden	0.06	
	Harrisonia perforata (Blanco) Merr. (AJPAC-257)	Khon tha	Root (ScanS)	Decoction (In)	Digestive System Disorders	Diarrhea	National park	0.06	

Appendix S1. (Continued)

Family	Scientific names (Vourcher no.)	Common Thai Name	Part used (Life form and Status)	Preparation/ Applications (Methods)	Health Disorders (Cook, 1995)	Symptoms and Ailments/ (Cook, 1995)	Source of Medicinal plant (threatened plants status)	UV	FIV
Rutaceae	*Naringi crenulata* (Roxb.) Nicolson (AJPAC-140)	Kra chae	Whole/Root/ Woody (ST)	Decoction (In)	Infections/ Infestations / Circulatory System Disorders/ Nutritional Disorders/	Fever/ Hemorrhoids/ Tonic	Community forest	0.2	
	Micromelum minutum (G.Forst.) Wight & Arn. (AJPAC-238)	Samat noi	Root (S/ST)	Grinding and drink (In) / Grinding and smear (Ext)	Infections/ Infestations /Poisonings/Injuries	Fever/Detoxicant /Abscess	Home garden / Community forest	0.2	
Sapindaceae	*Lepisanthes rubiginosa* (Roxb.) Leenh. (AJPAC-203)	Ma huat	Root (T)	Decoction (In)	Infections/ Infestations	Fever	Community forest	0.13	13
Simaroubaceae	*Ailanthus triphysa* (Dennst.) Alston (AJPAC-081)	Ma yom pa	Woody (T)	Decoction (In)	Muscular Skeletal System Disorders/	Pain	National park	0.13	33
	Brucea javanica (L.) Merr. (AJPAC-265)	Rat cha dat	Woody/Root/Leaf (S)	Decoction (In)	Digestive System Disorders	Flatulence	Community forest	0.06	
	Eurycoma longifolia Jack (AJPAC-260)	Pla lai phueak	Root (S/ST)	Decoction (In)/ Grinding and Smear (Ext)	Digestive System Disorders/ Circulatory System Disorders/ Nutritional Disorders/Skin/Subcutaneous Cellular Tissue Disorders	Gall stone/ Hemorrhoids/ Tonic/Shingles	Community forest	0.46	
Smilacaceae	*Smilax perfoliata* Lour. (AJPAC-088)	Kamlang khwai thuek	Root (C)	Decoction (In)	Nutritional Disorders	Tonic	Community forest	0.06	13
Solanaceae	*Cestrum nocturnum* L. (AJPAC-031)	Ra tri	Whole (ExS)	Decoction (In)	Circulatory System Disorders	Cardiopathy	Community forest	0.2	47

Appendix S1. (Continued)

Family	Scientific names (Vourcher no.)	Common Thai Name	Part used (Life form and Status)	Preparation/ Applications (Methods)	Health Disorders (Cook, 1995)	Symptoms and Ailments/ (Cook, 1995)	Source of Medicinal plant (threatened plants status)	UV	FIV
Solanaceae	*Solanum capsicoides* All. (AJPAC-011)	Ma khuea pro	Root/Fruit (S)	Decoction (In)/Soaking and Drink (In)	Respiratory System Disorders/ Inflammations/ Infection/Infestations	Coughs/ Mouth ulcers/ Chicken pok	Home garden	0.2	
Talinaceae	*Talinum paniculatum* (Jacq.) Gaertn. (AJPAC-122)	Som khon	Root (H)	Decoction (In)	Endocrine System Disorders /Circulatory System Disorders/ Infections/Infestations /	Anemia/Diabetes /Hypertension / Fever	Buy/Community forest	0.26	13
Tiliaceae	*Microcos tomentosa* Sm. (AJPAC-114)	Phlap phla	Woody (T)	Decoction (In)	Pregnancy/Birth/ Puerperium Disorders	Lactation	Community forest	0.13	6
Thymelaeaceae	Aquilaria crassna Pierre ex Lecomte (AJPAC-208)	Kritsana	Bark (T)	Inhale (In)	Respiratory System Disorders	Sinusitis	Community forest (R/CR)	0.13	13
	Enkleia malaccensis Griff. (AJPAC-252)	Po tao hai	Root (SC)	Decoction (In)	Digestive System Disorders	Diarrhea	Community forest	0.06	
Vitaceae	*Leea indica* (Burm.f.) Merr. (AJPAC-136)	Katang bai	Root/Leaf/Stem (S)	Decoction (In)	Digestive System Disorders/ Circulatory System Disorders	Gallstones/ Hemorrhoids	Community forest /National park	0.2	6
Zingiberaceae	*Alpinia galanga* (L.) Willd. (AJPAC-082)	Kha	Rhizome/Stem (ExH)	Decoction (In)/ Grinding and drink (In)	Digestive System Disorders/Muscular Skeletal System Disorders/	Flatulence/Pain	Home garden	0.4	93
	Catimbium speciosum (Wendl.) Holtt. (AJPAC-227)	Kha khom	Rhizome/Fruit (H)	Decoction (In)	Digestive System Disorders	Gastritis	Community forest	0.13	

Appendix S1. (Continued)

Family	Scientific names (Vourcher no.)	Common Thai Name	Part used (Life form and Status)	Preparation/ Applications (Methods)	Health Disorders (Cook, 1995)	Symptoms and Ailments/ (Cook, 1995)	Source of Medicinal plant (threatened plants status)	UV	FIV
Zingiberaceae	*Boesenbergia rotunda* (L.) Mansf. (AJPAC-246)	Kra chai	Tuber/Leaf (H)	Grinding and drink (In)/ Eat as fresh (Fd)	Skin/Subcutaneous Cellular Tissue Disorders	Allergy	Home garden	0.20	
	Curcuma aeruginosa Roxb. (AJPAC-100)	Wan maha mek	Rhizome (H)	Decoction (In)	Nutritional Disorders	Tonic	Home garden	0.06	
	Curcuma aromatica Salisb. (AJPAC-102)	Wan nang kham	Rhizome (H)	Grinding and Smear (Ext)/Decoctio n (In)	Genitourinary System Disorders /Digestive System Disorders/ Sensory System Disorders	Uterus conditions/ Flatulence/ Eye inflection	Home garden	0.2	
	Curcuma comosa Roxb. (AJPAC-104)	Wan chak mot luk	Rhizome (H)	Decoction (In)	Genitourinary System Disorders	Uterus conditions	Home garden	0.26	
	Curcuma longa L. (AJPAC-051)	Kha min	Rhizome (H)	Grinding and Smear (Ex)/Decoction (In)	Digestive System Disorders/ Skin/Subcutaneous Cellular Tissue Disorders/ Genitourinary System Disorders	Flatulence/ Allergy/Uterus conditions	Home garden	0.66	
	Kaempferia galanga L. (AJPAC-135)	Pro hom	Leaf/Rhizome (H)	Squeezing and drink (In)	Sensory System Disorders	Giddy	Community forest	0.06	
	Wurfbainia villosa (Lour.) Škorničk. & A.D.Poulsen (AJPAC-139)	Reo dong	Rhizome/Root (H)	Compress (Ext)/ Decoction (In)	Muscular Skeletal System Disorders	Pain	Home garden	0.26	
	Zingiber mekongense Gagnep. (AJPAC-126)	Phlai dam	Rhizome/Leaf (H)	Compress (Ext)	Muscular Skeletal System Disorders	Pain	Community forest/ Home garden	0.2	

Appendix S1. (Continued)

Family	Scientific names (Vourcher no.)	Common Thai Name	Part used (Life form and Status)	Preparation/ Applications (Methods)	Health Disorders (Cook, 1995)	Symptoms and Ailments/ (Cook, 1995)	Source of Medicinal plant (threatened plants status)	UV	FIV
Zingiberaceae	*Zingiber officinale* Roscoe (AJPAC-007)	Khing	Rhizome/Fruit/Flower/ Leaf (H)	Eat as fresh (Fd)/ Decoction (In)/ Grinding and drink (In)	Digestive System Disorders/Skin/ Subcutaneous Cellular Tissue Disorders	Flatulence / Allergy	Home garden	0.46	
	Zingiber purpureum Roscoe (AJPAC-009)	Phlai	Rhizome/Leaf (H)	Decoction (In)/ Eat as fresh (Fd)	Circulatory System Disorders /Digestive System Disorders	Haemagogue/ Flatulence	Home garden	0.46	
